# Preclinical Validation of the Heparin-Reactive Peptide p5+14 as a Molecular Imaging Agent for Visceral Amyloidosis

**DOI:** 10.3390/molecules20057657

**Published:** 2015-04-27

**Authors:** Jonathan S. Wall, Emily B. Martin, Tina Richey, Alan C. Stuckey, Sallie Macy, Craig Wooliver, Angela Williams, James S. Foster, Penney McWilliams-Koeppen, Ed Uberbacher, Xiaolin Cheng, Stephen J. Kennel

**Affiliations:** 1 Department of Medicine, University of Tennessee Graduate School of Medicine, Knoxville, TN 37920, USA; E-Mails: emartin@utmck.edu (E.B.M.); trichey@utmck.edu (T.R.); smacy@utmck.edu (S.M.); dwooliver@utmck.edu (C.W.); awilliam@utmck.edu (A.W.); jfoster3@uthsc.edu (J.S.F.); skennel@utmck.edu (S.J.K.); 2 Department of and Radiology, University of Tennessee Graduate School of Medicine, Knoxville, TN 37920, USA; E-Mails: astuckey@utmck.edu (A.C.S.); PMKoeppen@utmck.edu (P.M.-K.); 3 Bioscience Division, Oak Ridge National Laboratory, Oak Ridge, TN 37831, USA; E-Mails: uberbacherec@ornl.gov (E.U.); chengx@ornl.gov (X.C.); 4 Computer Science and Mathematics Division, Oak Ridge National Laboratory, Oak Ridge, TN 37831, USA

**Keywords:** glycosaminoglycans, amyloidosis, peptide, imaging, modeling, SPECT, p5+14

## Abstract

Amyloid is a complex pathologic matrix comprised principally of paracrystalline protein fibrils and heparan sulfate proteoglycans. Systemic amyloid diseases are rare, thus, routine diagnosis is often challenging. The glycosaminoglycans ubiquitously present in amyloid deposits are biochemically and electrochemically distinct from those found in the healthy tissues due to the high degree of sulfation. We have exploited this unique property and evaluated heparin-reactive peptides, such as p5+14, as novel agents for specifically targeting and imaging amyloid. Herein, we demonstrate that radiolabeled p5+14 effectively bound murine AA amyloid *in vivo* by using molecular imaging. Biotinylated peptide also reacted with the major forms of human amyloid in tissue sections as evidenced immunohistochemically. Furthermore, we have demonstrated that the peptide also binds synthetic amyloid fibrils that lack glycosaminoglycans implying that the dense anionic motif present on heparin is mimicked by the amyloid protein fibril itself. These biochemical and functional data support the translation of radiolabeled peptide p5+14 for the clinical imaging of amyloid in patients.

## 1. Introduction

Amyloidosis is a protein-folding disorder characterized by the aggregation and deposition of proteinaceous fibrils, associated with proteoglycans and serum-derived proteins, in vital organs and tissues [1,2,3]. The unrelenting accumulation of amyloid invariably leads to organ dysfunction and severe morbidity or death. The deposits can be cerebral, as in patients with Alzheimer’s, Huntington’s or prion diseases, or involve visceral organs as seen in patients with light chain (AL) and inflammation-associated (AA) amyloidoses [4,5]. The systemic amyloidoses are orphan disorders but account for ~3500 new patients annually in the US alone [6]. More than 28 proteins have been identified as constituents of fibrils in amyloid deposits. It is the nature of these proteins that differentiate the diseases, define the treatment regimen, and establish the prognosis.

The term amyloid (meaning “starch-like”) was coined in 1854 when a macroscopic brain tissue abnormality, stained with iodine, was shown to exhibit histochemical characteristics reminiscent of cellulose [7]. The glycosaminoglycans observed histochemically in amyloid deposits are a ubiquitous component of all amyloids regardless of the precursor protein from which the fibrils are formed or the anatomic site of deposition.

The major form of visceral amyloidosis is AL, a sporadic monoclonal plasma cell dyscrasia resulting in the deposition of fibrils composed of immunoglobulin light chain proteins with an estimated incidence of ~1.4 per 100,000 persons per year in the USA [6]. Systemic AA is associated with chronic inflammatory disorders such as arthritis, tuberculosis and familial Mediterranean fever [8,9]. Both AA and AL amyloidoses are associated with the accumulation of heparan sulfate proteoglycans (HSPG) at the site of amyloid deposition [10,11]. At present, there are no naturally occurring or experimental animal models that effectively recapitulate AL amyloidosis as seen in patients; however, several robust murine models of AA have been developed and proven invaluable for the study of amyloidogenesis and as platforms for the development of therapeutics [12,13,14].

The precise role of HSPG in the development and progression of amyloid disease is enigmatic but unequivocal [11,15,16,17,18,19]. In an experimental murine model of AA-amyloidosis, the up-regulation of HSPG synthesis was found to be spatially and temporally coincident with the deposition of AA-amyloid [15,16,19]. The precursor, sAA, possesses a well characterized 27-amino acid heparan sulfate binding motif [20], a structural feature shared by other amyloid-forming proteins, e.g., Aβ [21,22,23]. Thus, cellular HSPG may act as a nidus, or anchor, for precursor proteins thereby promoting the initiation of amyloid deposition. The critical role played by HS in the development of AA amyloidosis was exquisitely demonstrated using mice genetically engineered to overexpress the enzyme heparinase—an enzyme that cleaves heparan glycosaminoglycans [18]. In the absence of heparan sulfate, the mice were resistant to AA amyloid deposition following injection of pro-inflammatory stimuli—no deposits were observed in any tissue or organ, except the spleen where the transgene was ineffective due to the chimeric disposition of enzyme expression. Ultrastructural analysis of murine AA amyloid deposits, *in situ* and following standard isolation procedures, suggested that amyloid was a complex structure containing 3 nm-wide “ribbons” of chondroitin sulfate proteoglycan encompassed by a layer of HSPG forming 4.6 nm-wide strands. This macroscopic unit was decorated with the proteinaceous AA filaments (~2 nm in width) [13,24]. A similar multifaceted model for the amyloid matrix, comprising ordered, sulfated proteoglycan structures and fibrils, has been observed for other amyloids including β-amyloid associated amyloid (Aβ), AL, and those comprising β2-microglobulin (Aβ2M) and transthyretin (ATTR) [25,26,27], indicating a common role and uniform importance of proteoglycans in the development of amyloid pathology.

With respect to the development of specific amyloid-targeting agents, one of the most fundamental observations regarding amyloid-associated HSPG is that it is both biochemically and electrochemically distinct from the ubiquitous heparan sulfate (HS) found in healthy (amyloid-free) tissues. Lindahl and Lindahl demonstrated that HS derived from AA amyloid-laden liver, spleen, and kidney was similar in structure, but differed from HS derived from healthy tissues as evidenced by a shift in the disaccharide ratio [28]. Furthermore, the HSPG in murine AA amyloid can be specifically targeted by using the radio-iodinated single chain fragment variable antibody (scFv), NS4F5. This reagent was isolated from a phage library using human lung HS extract as the selection target [29]. The ^125^I-NS4F5 scFv specifically co-localized with the systemic AA amyloid deposits in mice and did not bind ligands in healthy tissues [30]. The precise saccharide ligand recognized by NS4F5 consists of HS with N-sulfation, C5-epimerization and high degrees of 2-*O*- and 6-*O*-sulfation [31]. Given that HS is invariably associated with amyloid and has a distinct hypersulfation pattern, akin to that of heparin, we have explored the use of synthetic heparin-binding peptides as specific amyloid-targeting agents for the purpose of generating novel molecular imaging agents for the detection of disease in patients.

Molecular imaging modalities, such as planar gamma scintigraphy, single photon emission computed tomography (SPECT) and positron emission tomography (PET), in conjunction with radiolabeled amyloid-reactive probes, have been used for the clinical detection of visceral amyloid, albeit not routinely. Currently, the gold-standard amyloid imaging agent, serum amyloid P component (^123^I-SAP), is routinely used for the clinical evaluation of whole-body amyloid load in numerous sites in Europe [32]; however, this agent is not approved by the Food and Drug Administration for use in the US likely due to its sensitivity to the stringent virus inactivation steps required by the agency. Other imaging agents have been evaluated for the clinical detection of visceral amyloid, including: bone-seeking phosphate derivatives, 3,3-disphosphono-1,2-propanodicarboxylic acid (DPD) and hydroxymethylene disphosphonate (HDP); proteins and antibodies, such as aprotinin, and 11-1F4; and the Aβ-imaging agents Vizamyl™ (aka, PiB analog) and Amyvid™ (aka, AV45); however, none of these efficiently detect systemic amyloid in all anatomic sites [33,34,35,36,37]. Therefore, in an attempt to develop a universal imaging agent for detecting systemic amyloidosis, we have evaluated radiolabeled heparin-binding peptides by using *ex vivo* amyloid-reactivity assays and amyloid imaging strategies [38,39,40].

We initially identified a peptide, p5, capable of quantitative, specific AA amyloid detection in mice [39]. Herein, we now describe preclinical validation studies for the related peptide, p5+14, an extended variant of p5 (Table 1), in support of potential translation and clinical evaluation as a specific amyloid-imaging agent.

**Table 1:** Physicochemical parameters of peptide p5+14.

Physical Property	
**Primary structure**	GGGYS KAQKA QAKQA KQAQK AQKAQ AKQAK QAQKA QKAQA KQAKQ
**Molecular weight**	4766.4 Da
**Number of amino acids**	45
**Theoretical pI**	10.74
**Net charge**	+12
**Lysine content**	26.7%
**Formula**	C_202_H_351_N_71_O_62_
**Predicted extinction coefficient (280 nm)**	1490 M^−1^·cm^−1^
**Predicted secondary structure**	α-helical

The additional 14 amino acids, including four additional positive charges (lysine residues), endowed peptide p5+14 with enhanced amyloid-binding properties (*i.e.*, increased affinity and extended amyloid reactivity). Longitudinal SPECT/CT imaging has been used to demonstrate the specific and long-term retention of radiolabeled p5+14 by AA amyloid-laden organs *in vivo*. In contrast, the peptide was rapidly “washed-out” from healthy tissues. Peptide p5+14 was further shown to bind specifically to human AL and ATTR amyloid-laden patient-derived tissue samples, by using histochemistry and autoradiography. Additionally, the underlying biochemical nature of the interaction of p5+14 with amyloid has, for the first time, been interrogated by using computer modelling. Together, our data demonstrate that hypersulfated heparin-like motifs associated with amyloid are an excellent biomarker for the pathology that can be efficiently targeted by p5+14, which may render this peptide an invaluable clinical tool for imaging amyloid burden in patients.

## 2. Results and Discussion

### 2.1. Structural Characteristics and Heparin Affinity of Peptide p5+14

**Figure 1:**
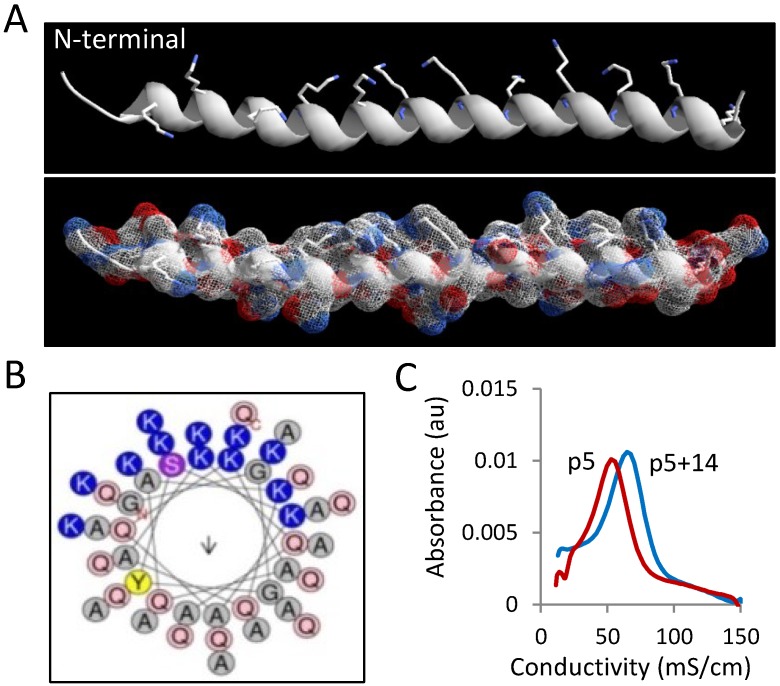
Peptide p5+14 is predicted to be α-helical and binds immobilized heparin. (**A**) The secondary structure of peptide p5+14 was predicted to be α-helical using the iTASSER algorithm [41]. The lysine side chains (blue) align along one surface of the helix; (**B**) Helical wheel representation of peptide p5+14 predicts linear array of lysine residues; (**C**) Peptide p5+14 (blue) bound immobilized heparin with greater affinity that the truncated peptide p5 (+8 net charge; red).

The 45-amino acid sequence of peptide p5+14 (Table 1) contains 12 lysine residues and a tyrosine residue at position 4, which is a site for facile radioiodination of the peptide, to allow for SPECT and PET molecular imaging studies. The sequence was submitted to the I-TASSER structure prediction server [41] which revealed that it likely adopted an α-helical secondary structure (Figure 1A). Due to the inherent heptad amino acid repeat (Lys-X-X-Lys-X-X-X, where X is Ala or Gln), the basic lysine side chains were predicted to be oriented along one face of the helix (Figure 1A). The helical wheel representation of p5+14 similarly predicted this configuration (Figure 1B). This secondary structure is similar to that predicted for the well-studied, amyloid-reactive peptide, p5 (a 31-amino acid truncated variant of p5+14 with a net charge of +8) [38,40]. Such a linear array of basic amino acid sidechains along the peptide helix is optimal for the binding of poly-basic peptides with heparin [42]. The relative affinity of p5+14 for immobilized heparin was determined chromatographically and compared to that found for the p5 peptide (Figure 1C). The 4 additional lysine residues in p5+14 resulted in a greater retention time as indicated by the shift in peak elution conductivity, *i.e.*, NaCl concentration required for peptide elution, from 53 mS/cm to 65 mS/cm for p5 *vs*. p5+14.

With respect to other parameters, peptide p5 exhibits similar amyloid binding properties in mice, as compared to p5+14 ([38] and data presented herein). However, the binding of p5+14 to synthetic amyloid fibrils, AA-laden mouse tissue homogenates, and human AL and ATTR amyloid extracts is greater (data not shown for p5). This invariably results from increased electrostatic interactions provided by the additional 4 lysine residues in peptide p5+14. Therefore, the enhanced affinity of peptide p5+14 for heparin relative to p5, in addition to greater solution stability, increased amyloid reactivity on human tissues and greater ease of radiolabeling with other clinically relevant isotopes, notably ^99m^Tc (Wall and Kennel, unpublished data), warranted *in vivo* investigations of this reagent as a specific amyloid-targeting peptide in mice. Longitudinal SPECT imaging and tissue biodistribution studies were designed to evaluate the specificity and longevity of the peptide-amyloid interaction *in vivo* and examine the off-target reactivity with healthy organs and tissues.

### 2.2. Longitudinal Biodistribution and SPECT/CT Imaging of ^125^I-p5+14 in Mice

**Figure 2:**
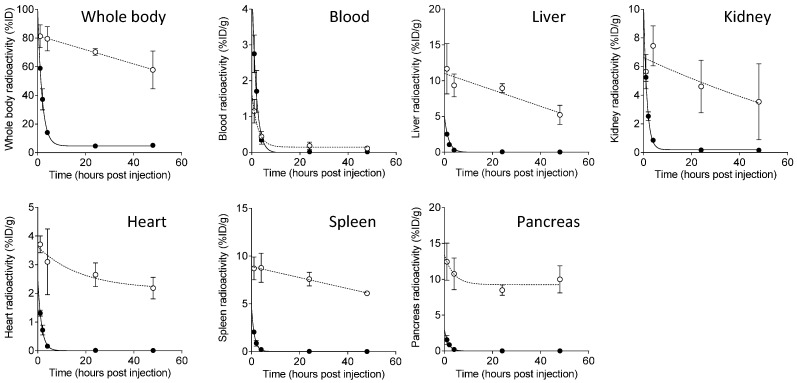
Whole body and organ retention of ^125^I-p5+14 in healthy and AA amyloid-laden mice. Whole body and tissue radioactivity measurements were made by gamma counting and expressed as % injected dose (%ID) or percent injected dose per gram of tissue (%ID/g) of wild type (closed circles) or mice with systemic AA (open circles) of mice injected. Data, expressed as mean ± SD were fitted using a single or double exponential decay. N = 3 mice for each time point.

Transgenic mice that constitutively express human interleukin-6 (e.g., H2/IL-6 mice) develop severe systemic AA due to the pro-inflammatory stimulus elicited by IL-6 [14]. H2/IL-6 mice, with AA amyloidosis, were injected with ^125^I-p5+14 to assess peptide biodistribution and the stability of binding in various tissues up to 48 h post injection (pi). Retention of radiolabeled peptide (percent injected dose [%ID] or per gram of tissue [%ID/g]) was measured in the whole animal as well as in the blood, liver, kidney, heart, spleen and pancreas (organs known to develop AA amyloid in this mouse model; Figure 2). At each time point there was enhanced retention of the ^125^I-p5+14 peptide in AA-laden tissues, and consequently the whole animal, as compared to WT mice, up to 48 h pi (Figure 2). The whole-body retention half-life of ^125^I-p5+14 was calculated to be ~1.5 h in WT mice, which contrasted dramatically with a >48 h half-life for mice with AA amyloidosis. Clearance of ^125^I-p5+14 from the blood was rapid in all mice. In healthy animals, the peptide was quickly cleared via renal excretion.

In contrast, in mice with AA, the peptide was retained, radiolabeled, in the visceral organs for up to 48 h pi. It is noteworthy that, in healthy mice, there was no significant retention of radioactivity at 2 h pi (<0.6%ID/g) in any organ demonstrating that the ^125^I-p5+14 peptide did not bind the sulfated proteoglycans present in healthy tissues. This is remarkable given the high density of HS in certain normal tissues, notably the hepatic sinusoids and renal glomerulae. The statistical deviation on each data point indicated that retention of the peptide was similar in all AA mice (*n* = 3, per time point) with the exception of the kidneys, which exhibited some variability—an observation likely due to heterogeneity in renal amyloid burden and residual renal function in diseased mice.

**Figure 3:**
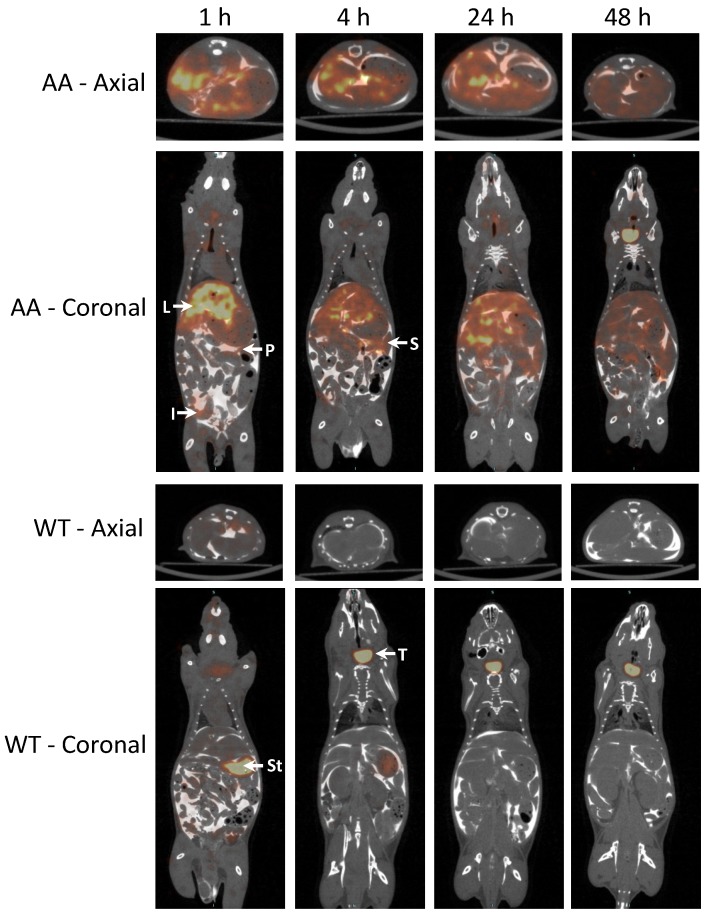
SPECT imaging of ^125^I-p5+14 revealed preferential uptake of peptide in amyloid-laden organs in AA mice, but not wild type (WT) animals. SPECT/CT images of mice (*n* = 3 per time point) injected with ^125^I-p5+14 were acquired at 1, 4, 24 and 48 h post-injection. Peptide was observed in the liver (L), spleen (S), pancreas (P) and intestines (I) of AA mice in axial and coronal views at all time points. In contrast, in WT mice, only free radioiodide liberated during catabolism was seen transiently in the stomach (St) and over the course of the investigation in the thyroid (T). Mice received, IP, ~300 µL of Iohexol CT contrast agent (1:1 in sterile PBS) 5 min prior to imaging to delineate abdominal organ boundaries.

Although there was loss of ^125^I-p5+14 over time in the visceral organs known to contain amyloid (notably, liver, spleen, pancreas, and heart), it remained sufficiently high to provide diagnostic whole-body SPECT/CT images of amyloid distribution even at 48 h pi (Figure 3). A comparison of SPECT/CT images from AA and WT mice revealed greater accumulation of ^125^I-p5+14 in the amyloid-laden spleen, liver and intestines at all time points. Dehalogenation of the radioiodinated p5+14, which occurs intracellularly in the renal proximal tubules (and to a lesser extent, hepatocytes) as a consequence of catabolism [43], liberated free radioiodide which was then sequestered by halide symporters in the stomach [44] and thyroid.

**Figure 4:**
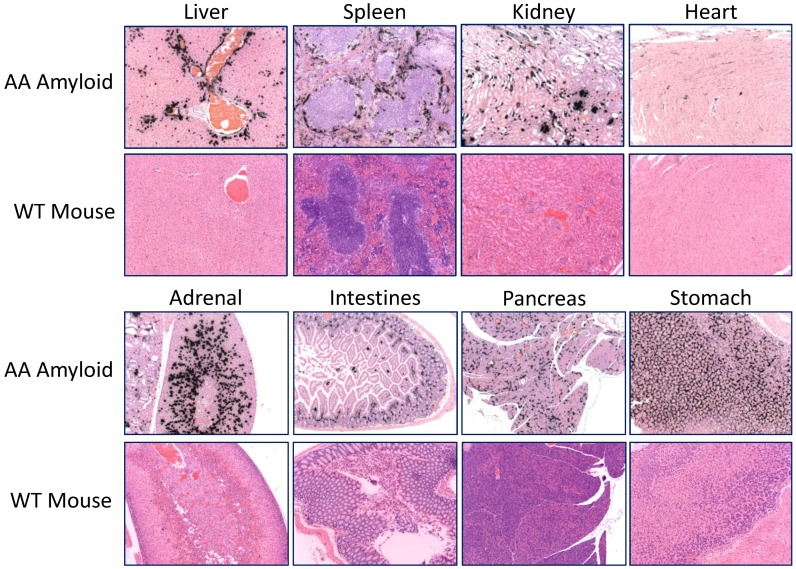
Microautoradiographic analysis of organs from AA and WT mice at 1 h pi revealed specific localization of ^125^I-p5+14 with tissue amyloid deposits. The binding of ^125^I-p5+14 with tissue amyloid in AA mice was evidenced as the appearance of black silver grains at the sites of amyloid deposition. No binding of the radiolabeled peptide was seen in amyloid-free WT mice. Original magnification, 10× objective.

This effect was more prominent in WT mice due to the fact that, in the absence of binding, all the ^125^I-p5+14 entered the catabolic pathway (Figure 4). The radioiodide entering the stomach forms hydroiodic acid (HI[^125^I]) which is released to the lumen of the stomach and is excreted rapidly (within ~4–8 h) via the intestines based on changes in the stomach radioactivity seen in the SPECT images. In contrast, radioiodide sequestered by the thyroid is organified (by the enzymatic iodination of thyroglobulin) and remains present in that tissue for more than 48 h (Figure 4).

SPECT imaging and biodistribution studies indicated that ^125^I-p5+14 was retained *in vivo* by organs known to contain amyloid and, in contrast, was rapidly “washed out” of healthy organs in WT mice. However, studies at the resolution of SPECT imaging (~1 mm spatial resolution) cannot confirm unequivocally that the organ-associated ^125^I-p5+14 was specifically associated amyloid deposits. To address this, micro-autoradiography was performed using 6 µm-thick sections of tissue harvested from the SPECT-imaged mice (Figure 4). This technique permitted the microscopic distribution of radioiodinated ^125^I-p5+14, as evidenced by the appearance of black silver grains, to be visualized with exquisite precision in formalin-fixed tissue sections. These data showed that the ^125^I-p5+14 peptide co-localized with amyloid in diseased tissues and was not retained by any cells or macroscopic structures in healthy tissues. The distribution of black silver grains in AA amyloid-laden tissues coincided precisely with the amyloid as seen in consecutive sections stained with Congo red.

The extensive systemic uptake of ^125^I-p5+14 in amyloid seen in this panel of tissues was reminiscent of the distribution of radiolabeled SAP, which, with the exception of pancreatic AA amyloid, binds AA deposits in all anatomic sites in this mouse model [45,46]. SPECT imaging using peptide p5+14 was superior to the bone-seeking and Aβ amyloid imaging agents, which have a limited distribution *in vivo* and only effectively image cardiac amyloidosis [36,37]. The lack of peptide uptake by WT mice confirmed that the amyloid-associated ligand recognized by ^125^I-p5+14 was not expressed in healthy tissues. The preferential binding of peptide with pathologic deposits supports our contention that radiolabeled p5+14 peptide may afford a specific and sensitive agent for the detection of amyloidosis in patients. 

**Figure 5:**
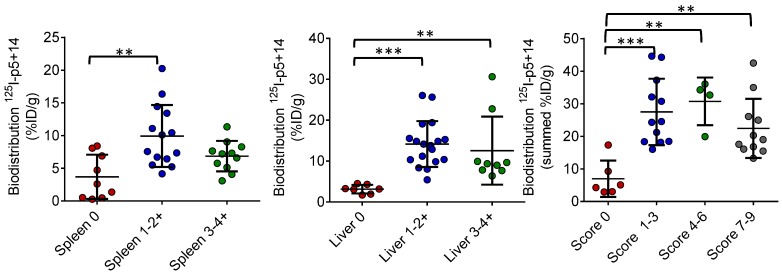
Accumulation of ^125^I-p5+14 in AA amyloid laden spleen, liver, and whole body increases with amyloid load. The amyloid load in the spleen, liver and whole body (the sum of liver, spleen and heart) was estimated qualitatively (0 to 4+) based on the area of birefringent amyloid seen in Congo red-stained tissue sections and compared to the uptake of ^125^I-p5+14 (%ID/g). Each point represents an individual mouse. Bars denote mean ± SD of the binned samples. Comparisons were made using a one-way ANOVA with multiple comparisons, with Tukey’s range test, where, ** *p* < 0.005, *** *p* < 0.0001.

We next sought to determine whether the accumulation of peptide in affected organs correlated quantitatively with the amyloid load. This is an important feature of an imaging agent if quantitative detection of amyloid is required, as it would be for monitoring response to therapy. Therefore, the amount of radiolabeled peptide in the liver, spleen, and “whole body” (the sum of scores for the liver, spleen and heart; %ID/g) was compared to a qualitative measure of amyloid burden based on scoring of the Congo red-stained sections (0 to 4+). The Congo red scores for the liver and spleen, the two organs with greatest AA amyloid deposition in the mice (*n* = 34), were binned into 0 to 2+ (mild to moderate) or 3 to 4+ (severe) amyloid cases and compared to the peptide biodistribution (Figure 5). Analysis of this large cohort of mice revealed a significant difference between the tissue uptake of ^125^I-p5+14 in amyloid free organs (based on Congo red histochemistry) as compared to those with mild or severe AA amyloidosis (Figure 5).

Interestingly, there was evidence of significant ^125^I-p5+14 retention (>3%ID/g) in the spleen, of mice deemed to be amyloid-free based on histochemical staining (Figure 5, left panel). This apparent false-positive uptake of peptide may be due to the fact that, since the induction of perlecan is one of the earliest events in AA amyloid deposition in mice [15], p5+14 reactivity may precede the appearance of Congo red-birefringent amyloid fibrils. These data also indicated that there was no significant difference in the uptake of ^125^I-p5+14 between mice with mild/moderate amyloid and mice with severe disease (Figure 5). This phenomenon has been previously reported for high-affinity amyloid reactive peptides binding to extensive amyloid deposits [40] and likely results from the inability of amyloidophilic radiotracers to penetrate significantly the extensive waxy deposits that develop in the AA mouse model. This could be a function of the short half-life of the peptide in circulation and the vast amyloid beds that form in the mouse. In humans, we anticipate that the circulating half-life of the peptide would be longer than that seen in mice, due to their slower metabolism; furthermore, the amyloid in patients is generally less extensive as compared to that seen in the AA mouse model. Thus, we believe that the apparent inability of the peptide to discern “2+” from “4+” amyloid in mice would not compromise the use of this reagent for the detection of early amyloid disease in patients, which is the greatest clinical need.

### 2.3. Pharmacokinetic Analysis of ^124^I-p5+14 in WT Mice: A PET/CT Study

**Figure 6:**
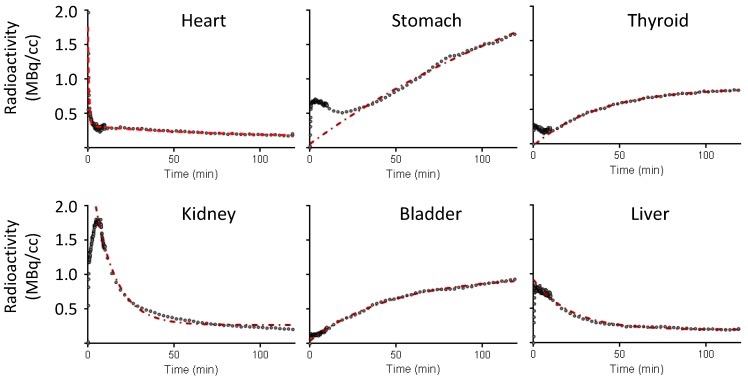
Pharmacokinetics of ^124^I-p5+14 in WT mice over 2 h post-injection. Regions of interest were drawn on the PET data over the heart (blood pool), stomach, thyroid, kidney, bladder and liver. Time activity curves (TAC) were generated using Inveon Research Workplace software analysis package (filled circles). Exponential equations were used to quantify the rates of accumulation or loss of radioactivity in each tissue (red dashed line, Table 2). Goodness of fit (R^2^) was >0.9 for all data.

Peptide p5+14 was shown to be rapidly excreted from WT mice, based on the 48-h longitudinal biodistribution study (Figure 2). Due to the limited temporal resolution of these data the pharmacokinetics (PK) of ^124^I-labeled peptide were followed over the initial 2 h pi by using dynamic PET imaging. Dynamic PET data were acquired in WT mice and regions of interest were drawn on the reconstructed images to represent the heart (a surrogate for blood pool), stomach, thyroid, kidney, bladder and liver—all potential sites of peptide catabolism and radioiodide sequestration (Figure 6). Analysis of the generated time activity curves (TAC) indicated that ^124^I-p5+14 was rapidly cleared from the blood pool (heart) with bi-exponential kinetics (Table 2). 

**Table 2:** Pharmacokinetic rate constants for ^124^I-p5+14 in amyloid free, wild type mice.

Organ	Fitting Eqn.	Goodness (R^2^)	Rate 1 (fast) ^3^	Rate 2 (slow)	T_1/2_ Rate 1 ^4^	T_1/2_ Rate 2
**Bladder**	1 Exp. ^1^	0.997	0.02085	na	33.24	na
**Stomach**	1 Exp.	0.991	0.00528	na	131.30	na
**Thyroid**	1 Exp.	0.999	0.02609	na	26.57	na
**Kidney**	1 Exp.	0.993	0.08022	na	8.64	na
**Liver**	1 Exp.	0.988	0.04616	na	15.02	na
**Heart**	2 Exp. ^2^	0.944	1.832	0.01403	0.39	49.41

^1^ Single exponential equation; ^2^ Double exponential equation; ^3^ Rate 1 and 2 expressed in min^−1^; ^4^ T_1/2_ is ln2/K, expressed in min.

The t_max_ for the blood was ~20 sec pi with a half-life for the fast rate of ~24 sec indicating very fast blood clearance (Table 2). The peptide was catabolized predominantly via renal excretion (although hepatobiliary clearance may be involved to a lesser degree), as evidenced by the intense accumulation of radioactivity in the kidney (t_max_ ~7 min) that decreased exponentially (t_1/2_ ~9 min), due to both passage of ^124^I-p5+14 into the urinary bladder and dehalogenation by cytoplasmic Type 1 deiodinases in renal tubules (Figure 6) [43]. As previously observed for related peptides [38], the liberated radioiodide entered the circulation and was sequestered by the thyroid and stomach, as evidenced by the increase in radioactivity after lag times of 11 and 18 min, respectively (Figure 6).

These data further confirm that p5+14 was rapidly cleared from healthy organs, (within 2 h pi) and did not bind the ubiquitous cell- or extracellular matrix-associated HSPG, presumably due to the relatively low sulfation content of the proteoglycans in these tissues.

In terms of potential translation of peptide p5+14 as a visceral amyloid imaging agent, our observations that there was scant retention of the reagent in healthy tissues and rapid dehalogenation of unbound tracer in the kidney, are of tremendous benefit. These properties will permit accurate imaging of renal amyloid disease if imaging is performed at time points when only amyloid-bound peptide (which is not prone to rapid dehalogenation, see Figure 2) remains radiolabeled. Furthermore, dehalogenation of peptide not bound to amyloid will reduce the radiation dose to healthy organs in imaged subjects. 

### 2.4. Pan-Amyloid Reactivity of Peptide p5+14

**Figure 7:**
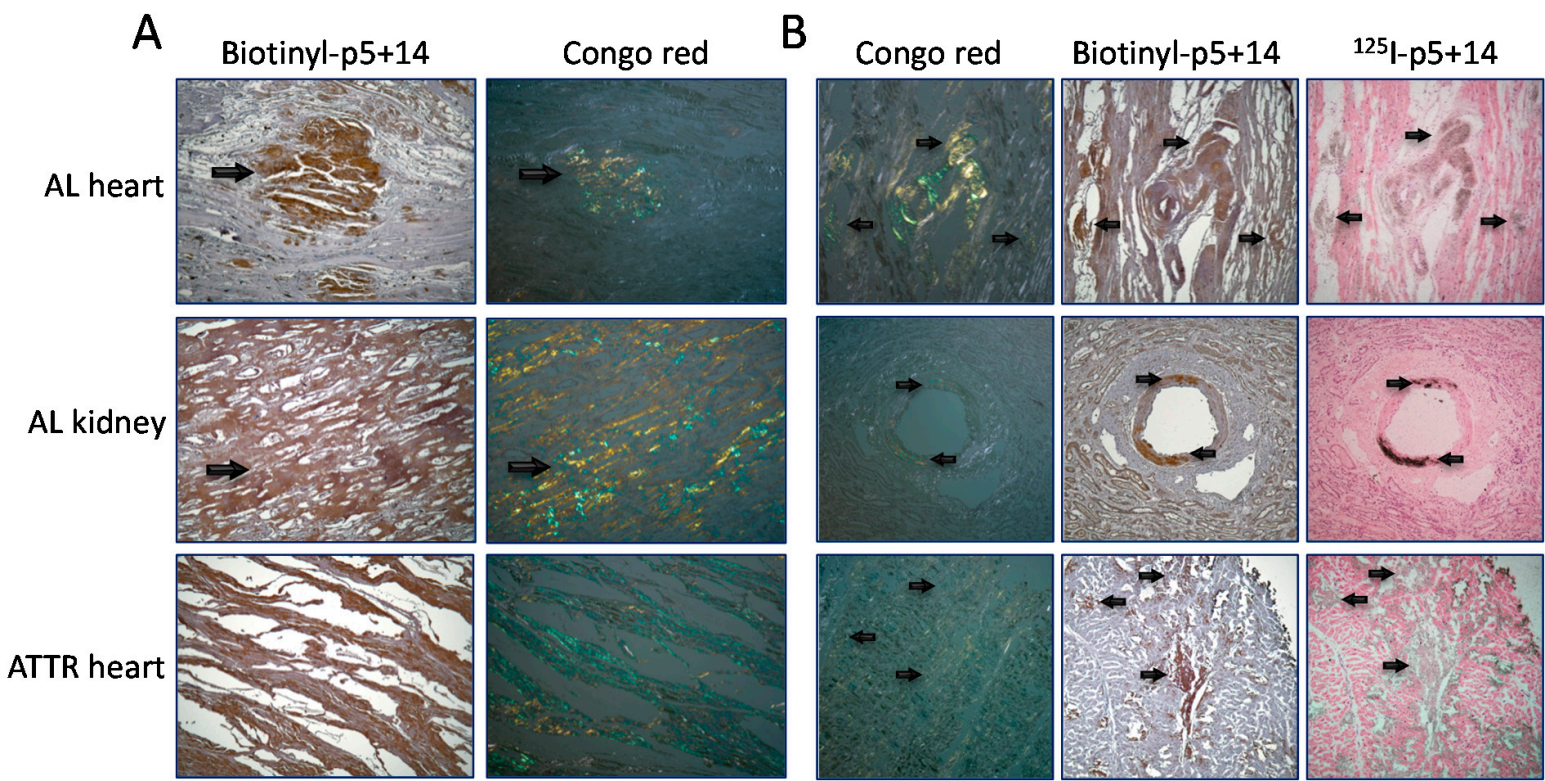
Peptide p5+14 specifically binds human AL and ATTR amyloid in tissue sections. (**A**) Biotinylated p5+14 (brown) stained human renal and cardiac AL, or cardiac ATTR amyloid seen as green-gold birefringence in Congo red-stained sections; (**B**) ^125^I-p5+14 binding correlated with the distribution of amyloid in tissue sections seen using biotinylated p5+14 (brown) and Congo red birefringence (green gold). Arrows indicate the sites of amyloid in Congo red section and peptide p5+14 accumulations.

The amyloid-targeting efficacy of peptide p5+14 has been demonstrated *in vivo* using a panel of imaging and biodistribution studies in a murine model of AA systemic amyloidosis; however, AA is not a major form of the disease in man. Therefore, we sought to assess the reactivity of peptide with the two major human forms, AL and ATTR. Due to the lack of experimental or naturally occurring animal models of systemic AL and ATTR, we used peptide-histochemistry (with biotinylated p5+14) and micro-autoradiography (with ^125^I-p5+14) to study p5+14 binding to human AL or ATTR in formalin-fixed paraffin-embedded (FFPE) tissue sections (Figure 7).

To confirm the amyloid distribution in each tissue, consecutive sections were stained with Congo red and viewed under cross-polarized illumination. The appearance of biotinylated p5+14, evidenced by brown diaminobenzidene staining (arrows), correlated precisely with the presence of amyloid seen as green-gold birefringent deposits in the Congo red-stained section (arrow; Figure 7A). Similarly, the radioiodinated p5+14, seen as diffuse black silver grains, co-localized with both AL and ATTR amyloid (arrows) in all samples (Figure 7B). These data indicated that p5+14 specifically bound the major forms of visceral amyloid found in man, AL and ATTR. Similar multi-amyloid reactivity has been previously described for other reagents including monoclonal antibodies and proteins (e.g., B10, WO1, 2A4, 11-1F4, and SAP [47,48,49,50,51]) and small molecules (e.g., Vizamyl™ and Amyvid™, Congo red, and thioflavin T [52,53]). Many of these reagents are used, or are being evaluated for use, as diagnostic or therapeutic agents for patients with amyloidosis.

To explore the amyloid-peptide interaction quantitatively, human AL and ATTR amyloid extracts were studied in an *in vitro* peptide binding assay. In this system, AL and ATTR amyloid extracts, containing insoluble proteoglycans and fibrils, were incubated with ^125^I-p5+14 and the binding of radiolabeled peptide determined (Table 3). Samples obtained from 10 patients diagnosed with ALκ or ALλ isolated from autopsy-derived liver or spleen tissues, as well as one cardiac ATTR extract, were analyzed in this assay. With singular exception (λ2 AL10), ^125^I-p5+14 bound the amyloid extracts >50% (non-specific binding was ~10% for this peptide to healthy tissue homogenates—data not shown).

**Table 3:** Binding of ^125^I-p5+14 to human AL and ATTR amyloid extracts.

Human Amyloid Extracts	% Bound (mean ± SD)
**ATTR heart**	79.93 ± 1.23
**κ4 AL01 spleen**	97.00 ± 0.11
**λ2 AL02 liver**	92.95 ± 0.31
**λ2 AL02 spleen**	96.76 ± 3.65
**λ1 AL03 liver**	91.16 ± 0.87
**λ3 AL04 spleen**	92.29 ± 0.30
**λ3 AL04 liver**	88.44 ± 0.25
**λ4 AL05 spleen**	95.44 ± 0.03
**λ2 AL06 liver**	89.66 ± 0.91
**λ2 AL06 spleen**	97.11 ± 0.05
**λ1 AL07 liver**	51.81 ± 0.25
**κ1 AL08 liver**	96.35 ± 0.47
**λ2 AL09 liver**	84.99 ± 0.14
**λ2 AL10 spleen**	12.25 ± 1.08

% bound represents the amount of peptide bound to the substrate after a 1 h incubation. N = 2 for each assay.

The peptide-amyloid reactivity varied amongst the test samples with a mean ± SD and median % bound of 91% ± 24% and 92%, respectively. In five of the 13 samples, intense binding (>95%) was observed; in only one sample, λ2 AL10, was <50% peptide bound (Table 3). The broad distribution in binding, reflected in the large SD, reflects the heterogeneity of amyloid composition with respect to the amount of HSPG and fibril. Regardless, this study contributed unequivocal evidence of peptide reactivity with both human AL and ATTR amyloid.

Our initial hypothesis that p5+14 binds amyloid via the GAG moieties is based on the observed binding to heparin, and the presence of heparin-like proteoglycans in the deposits. However, we have previously reported that the p5 peptide was reactive with synthetic AL fibrils devoid of HSPGs [54]. The underlying molecular mechanism for this interaction was not fully explored. Therefore, we similarly evaluated the interaction of ^125^I-p5+14 with a preparation of synthetic amyloid fibrils composed of a recombinant human λ6 variable domain derived from the sequence of an amyloidogenic LC isolated from patient Wil (rVλ6Wil; [55]).

The peptide bound HSPG-free synthetic rVλ6Wil fibrils, in the *ex vivo* binding assay, with similar avidity as compared to the human AL amyloid extracts (95.7% bound). The implications from this observation are ostensibly two-fold. First, the binding of p5+14 with amyloid *in vivo*, in tissue or as extracts probably involved interactions with the hypersulfated GAGs as well as the protein fibrils—either with equivalent avidity or, more likely, preferentially to one of those components. Secondly, amyloid fibrils and heparin must share a structurally homologous, or common, electrostatic motif—a collective phenotype recognized by p5+14. These observations predict that other entities that bind amyloid fibrils may also interact with highly sulfated GAG moieties. Such cross-reactivity has been observed for both the receptor for advanced glycation endproducts (RAGE), a fibril-binding membrane receptor which also binds chondroitin, dermatan, and heparan sulfate [56], and the amyloid-reactive camelid antibody B10 which binds fibrils, heparin, and the polyanionic surface of DNA via electrostatic interactions [47]. Based on these observations, a motif, presumably ionic in nature, is common to both amyloid fibrils and the hypersulfated amyloid-associated HSPGs, which may greatly enhance the reactivity of p5+14 with amyloid and may provide uniformly avid binding to the heterogeneous deposits.

### 2.5. Peptide p5+14 Binds Amyloid Fibrils via Electrostatic Interactions

**Figure 8:**
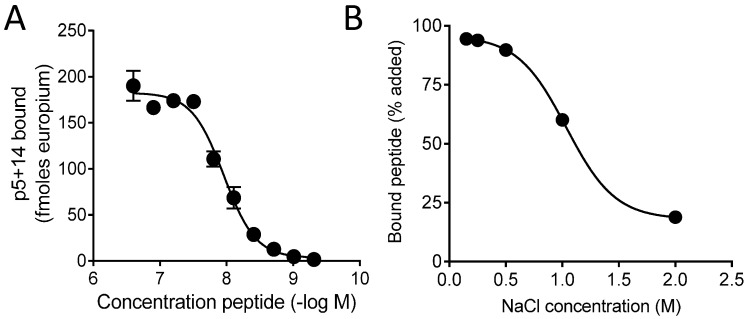
Peptide p5+14 avidly bound synthetic rVλ6Wil fibrils via electrostatic interactions. (**A**) Titration of biotinylated p5+14 on immobilized rVλ6Wil fibrils. Each point is the mean ± SD, *n* = 3 replicates; (**B**) Binding of ^125^I-p5+14 to rVλ6Wil fibrils in suspension (bound peptide expressed as % total counts added) decreased as the ionic strength increased to 2 M. Data were fit to a sigmoidal equation and are mean ± SD, *n* = 2 replicates.

To further explore the observed interaction of peptide p5+14 with synthetic fibrils, we performed binding studies in the presence and absence of high salt (Figure 8). Titration of rVλ6Wil fibrils with biotinylated p5+14 yielded saturable binding with an EC_50_ (half maximal binding) of ~15 nM (Figure 8A). The binding was shown to be dominated by electrostatic interactions and was perturbed by increasing the ionic strength of the aqueous environs (Figure 8B). The amount of bound p5+14 reduced from 95% to ~20% as the NaCl concentration increased to 2 M. The EC_50_ for this interaction was ~1.0 M NaCl, indicating an avid electrostatic binding between peptide and fibril.

**Figure 9:**
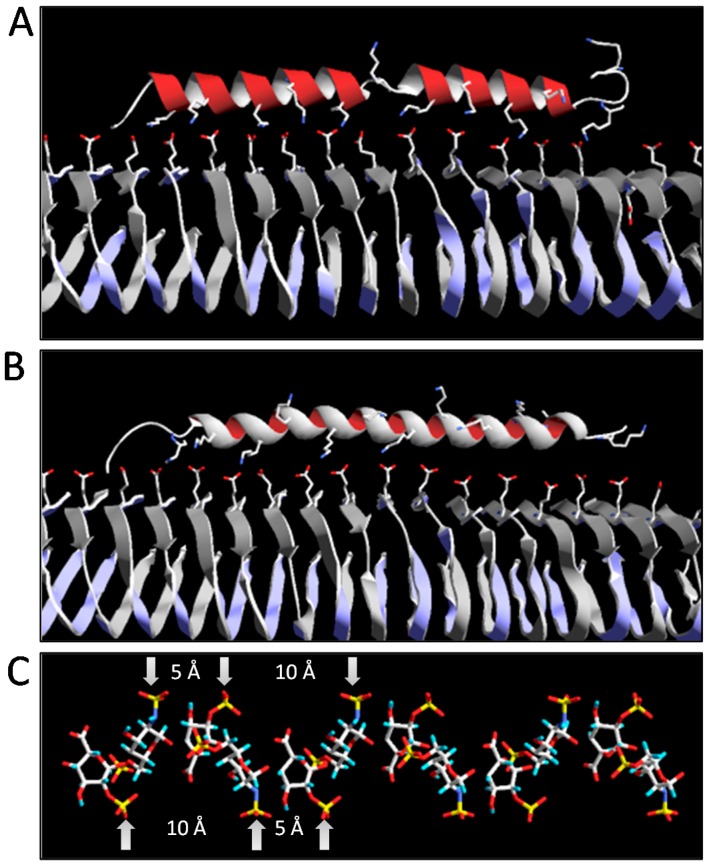
Molecular dynamic simulation of peptide p5+14 with an archetypal Aβ(17-42) amyloid fibril shows the dependence of electrostatic interactions. (**A**) Binding of peptide p5+14 (red helix) to the Aβ fibrils (blue beta strands; PDB# 1BEG), in a milieu of 0.1 M ionic strength, involves interactions between lysine sidechains of the peptide (blue) and the glutamate 22 side chains of the arrayed Aβ monomers (red); (**B**) In a medium of 1.0 M ionic strength, less side-chain interactions are observed; (**C**) The structure of heparin (PDB# 1HPN) with linear spacing of charged moieties with a 5 Å or 10 Å periodicity.

Further insight into the precise molecular interactions underlying the peptide-fibril interaction was achieved by using computer modeling. Unfortunately, there are, at present, no suitable models of AL amyloid fibrils that could be used as a substrate in these studies. The most widely accepted models of amyloid fibrils are those composed of the Alzheimer’s disease-associated Aβ peptide [57,58]. Given the multi-amyloid reactivity of p5+14 peptide, we deemed it reasonable to use the Aβ fibril model as an exemplar for all amyloid fibrils. Therefore, molecular dynamic simulations were performed using the α-helical conformation of p5+14 and an Aβ(17-42) fibril model in a milieu of increasing ionic strength (Figure 9).

As predicted by the *ex vivo* binding assay, the modeled interactions of p5+14 with the Aβ fibril were dominated by electrostatic interactions between the linear array of lysine side chains on the peptide (red helix, Figure 9A) and a glutamate at position 22 in the Aβ peptide (blue β-sheet array, Figure 9A) that is presented in a linear array on the outer surface of the monomers comprising the fibril. In a milieu of 1 M ionic strength (Figure 9B), the side chain interactions were still present but, as expected, were greatly diminished by the presence of high concentrations of counterion (Figure 9B). Binding energy calculations confirmed that electrostatic interactions contributed significantly to the binding of the p5+14 peptide to the Aβ fibril (Table 4). The free energy of the electrostatic component of p5+14 binding to Aβ fibril decreased from −81 to −31 kcal/mol as the ionic strength increased; consequently, the total binding free energy decreased by ~30% from −157 kcal/mol in 0 M NaCl (Table 4).

A rudimentary analysis of the spacing of the exposed charged amino acid sidechains in the fibril, the peptide, and the sulfate groups of heparin (Figure 9C; PDB# 1hpn) revealed a relatively consistent spacing of ~4.5 Å, ~6 Å, and 5 or 10 Å, for the glutamate 22, lysine side chains, and heparin sulfate groups, respectively. The parity in the distribution of charged motifs along these three polymers undoubtedly underlies the ability of peptide p5+14, and similar reagents, to bind heparin, hypersulfated HS, fibrils and other polyanionic substrates [40,47,54]. Although this binding simulation has provided a rationale for the reactivity of peptide p5+14 with various anionic polymers, we note that the Aβ fibril structure used may not accurately represent the structural complexity of fibril morphologies seen *in vivo* (or even *in vitro*) [59]. However, a linear array of β-peptides with uniform spacing has also been proposed for fibrils composed of islet amyloid polypeptide [60]; thus, the Aβ fibril provides an appropriate archetype for these preliminary investigations.

**Table 4:** Binding energies of the p5+14 peptide to the Aβ(17-42) fibril computed using the MM-PB/SA method.

Ionic Concentration	Δ*E_VDW_*	Δ*G_PB,elec_*	Δ*G_nonpolar_*	Δ*G_bind_*
**0 M**	−66.0 ± 8.4	−81.3 ± 9.1	1407.3 ± 92.6	−157.5
**0.1 M**	−66.0 ± 8.4	−42.8 ± 8.8	1407.3 ± 92.6	−119.0
**1.0 M**	−66.0 ± 8.4	−31.4 ± 8.6	1407.3 ± 92.6	−107.6

Δ*E_VDW_* is the van der Waals contributions, whereas Δ*G_nonpolar_* is the nonpolar component of the solvation free energy estimated by surface area. Δ*G_PB,elec_* is the electrostatic component of the binding energy Δ*G_bind_*. All values are in kcal/mol.

The data presented herein provide a mechanistic view of p5+14 binding to amyloid and extend our original hypothesis that the peptide bound exclusively the hypersulfated GAG components of the deposits. Given the expanded reactivity of the peptide, it is remarkable that there is no retention by cells or basement membrane structures in healthy, amyloid-free organs indicating that highly charged anionic polymers have restricted distribution in mammals and may well be present only as heparin, nucleic acids, pathologic amyloid fibrils, and the hypersulfated amyloid-associated GAGs. The potential for multi-ligand, yet specific, binding strengthens the value of this peptide as a pan-amyloid-targeting reagent *in vivo* that may be useful for the non-invasive, quantitative detection of amyloid in patients.

## 3. Experimental Section

### 3.1. Ethics Statement

All animal studies described herein were carried out in accordance with protocols approved by the University of Tennessee Institutional Animal Care and Use Committee and in accordance with the guidelines provided by Office of Laboratory Animal Welfare (OLAW) and the Guide for the Care and Use of Laboratory Animals. The University of Tennessee Graduate School of Medicine is a AAALAC-I-accredited institution.

### 3.2. Peptide Preparation

Peptide p5+14 was purchased from Keck Laboratories (New Haven, CT, USA) as an ~80% pure preparation and further purified by RP-HPLC (1100 series, Agilent, Santa Clara, CA, USA) by using a C3 solid phase and with a linear gradient of 0% to 50% acetonitrile in water with 0.05% v/v trifluoroacetic acid (flow rate of 1 mL/min) mobile phase. One mL-fractions were collected, pooled, and the integrity of the peptide was verified by mass spectrometry. The rVλ6Wil protein was expressed in *E. coli* and isolated from the periplasmic space, as previously described [55].

### 3.3. Murine Model of AA Amyloidosis

Systemic visceral AA amyloidosis was induced in H2-Ld-huIL-6 Tg Balb/c transgenic mice that constitutively express the human interleukin-6 transgene [14,61] by iv injection of 10 µg of purified, splenic AA amyloid (amyloid enhancing factor; AEF) [62] in 100 µL of sterile phosphate-buffered saline (PBS). Peptide was evaluated in mice at 4–6 wk post AEF injection when amyloid load was significant.

### 3.4. Solid Phase Heparin Binding Studies

Binding to solid-phase heparin was performed with a 20 mL bed-volume Heparin FF16/10 column (#28-9365-49, GE Healthcare Life Sciences, Piscataway, NJ, USA) using a Biologic duoflow FPLC system (BioRad, Hercules, CA, USA). An aliquot of 0.5 mg of peptide (1 mg/mL) was injected over 30 s and eluted using a linear gradient, from 0–2.0 M NaCl, over 10 min with a flow rate of 2 mL/min. The absorbance at 230 nm and the conductivity (mS/cm) were measured using in-line detectors. The elution profile of p5+14 was compared to the previously reported data for the peptide p5 [40].

### 3.5. Radiotracer Preparation

For serial SPECT/CT imaging in cohorts (*n* = 3) of AA and healthy wild type (WT) mice, peptide p5+14 (100 µg) was radioiodinated with ~4 mCi of ^125^I (Perkin Elmer, Waltham, MA, USA), using 20 µg chloramine T as the oxidizing agent [63]. After quenching the reaction with 20 µg sodium metabisulfite, the radiolabeled peptide was diluted into 0.1% sterile gelatin in PBS and free radioiodide removed by size exclusion chromatography on a 5 mL PD10 (GE Healthcare) solid phase equilibrated with 0.1% gelatin/PBS. Fractions 0f 200 µL were collected and those containing the maximal radioactivity (indicative of ^125^I-p5+14) were pooled, and the product’s radiochemical purity was established by SDS polyacrylamide gel electrophoresis (PAGE) analyzed by phosphor imaging (Cyclone Storage Phosphor System, Perkin Elmer, Shelton, CT, USA).

Dynamic PET imaging required radioiodination of the peptide with positron emitter, Iodine-124 (^124^I; IBA Molecular, Sterling, WV, USA). Peptide p5+14 was radiolabeled using the Iodogen (Piercechemical, Rockford, IL, USA) oxidation method, as described [38]. Briefly, 10 µL of 0.5 M NaPO4 buffer pH 7.6 was added to a 1.5 mL microcentrifuge tube followed by addition of ~50 µL of ^124^I (~2 mCi). One µL of 2 mg/mL fresh ascorbic acid solution in phosphate buffered saline (PBS) was added to ensure complete reduction of ^124^I-iodine. Approximately 25 µL (~50 µg) of peptide dissolved in distilled H2O was mixed with ~75 µL of PBS and added to the reaction mixture. Seven µL of 1 mg/mL Iodogen dissolved in acetonitrile was added to initiate the reaction. After 2 min, the reaction was quenched by addition of 50 µL of 2 mg/mL ascorbic acid. Twenty µL of blue dextran and 50 µL of filtered (0.2 µm pore sized) 0.1% gelatin in PBS were added to the reaction mixture. Radiolabeled product was separated from free ^124^I by size exclusion chromatography as described above. Fractions were manually collected as the blue dextran marker reached the base of the column and the radioactivity in each measured using a dose calibrator (Capintec, Model 15, Ramsey, NJ, USA) set for ^124^I detection. Peak fractions were pooled for use in the study, and radiopurity was assessed as described above.

### 3.6. Longitudinal SPECT/CT Imaging of ^125^I-p5+14 in AA and WT Mice

Imaging was performed as described [46]. Briefly, WT or AA amyloid mice (*n* = 3) were injected with ~3 µg of ^125^I-p5+14, 125 µCi (specific activity ~40 µCi/µg) in the lateral tail vein. After the appropriate uptake time (1, 4, 24, and 48 h), mice were euthanized by isoflurane inhalation overdose. SPECT images were acquired using an Inveon trimodality imaging platform (Siemens Preclinical Solution, Knoxville, TN, USA; [64]) running Inveon Acquisition Workplace software (ver. 2.0). Low energy (^125^I; 25–45 keV) gamma photons were acquired at each of 60, 16-sec projections with 90 mm of bed travel. A 1 mm-diameter, 5-pinhole (Mouse Whole Body) collimator was used at 30 mm from the center of the field of view. Data were reconstructed post hoc onto an 88 × 88 × 312 matrix with isotropic 0.50 mm voxels using a 3D ordered subset expectation maximization (OSEM) algorithm (eight iterations; six subsets).

CT data were acquired using an x-ray voltage biased to 80 kVp with a 500 mA anode current, with 4 × 4 binning. A 225 msec exposure was used, and 360, 1-degree projections were collected. The data were reconstructed using an implementation of the Feldkamp filtered back-projection algorithm [65] onto a 512 × 512 × 1296 matrix with isotropic 0.106 mm voxels. SPECT and CT datasets were automatically co-registered and visualized by using the Inveon Research Workplace visualization software package (Siemens Preclinical Solution). Mice were administered, IP, ~300 µL of Iohexol CT contrast agent diluted 1:1 in sterile PBS, 5 min before the imaging data were acquired.

### 3.7. Dynamic Biodistribution of ^124^I-p5+14 in WT Mice Using PET/CT Imaging

The procedure for dynamic PET imaging has been reported [38]. Briefly, mice were anesthetized in an induction chamber by exposure to 3% isoflurane in oxygen before being maintained with 2% isoflurane administered via a nose-cone for placement of an IV catheter in the lateral tail vein. Anesthetized mice were positioned in a heated imaging chamber (M2M, Cleveland, OH, USA). PET/CT data were acquired using the Inveon trimodality platform (Siemens Preclinical Solutions). Dynamic PET data acquisition was initiated ~5 s prior to injection of the radiotracer. The mice received ~5 µg of ^124^I-p5+14 (~250 µCi; specific activity ~50 µCi/µg) in ~200 µL 0.1% gelatin/PBS solution with 1 mM ethylenediaminetetraacetic acid (to prevent occlusion of the indwelling catheter). The PET data were acquired for 2 h and then histogrammed for ^124^I. The data were reconstructed into the following time bins: 10 × 1 s, 10 × 30 s, 30 × 114 s, and 60 frames covering the remainder of the data. The PET data were automatically corrected for attenuation and scatter by using the CT dataset. PET data were reconstructed using a 3D OSEM with maximum *a posteriori* algorithm. The image matrix was 256 × 256 × 159 with a reconstructed pixel size of 0.0215 × 0.0215 × 0.796 mm.

### 3.8. Biodistribution Measurements

Samples of liver, spleen, pancreas, kidneys, small and large intestines, stomach and heart were harvested post mortem from every mouse undergoing imaging with ^125^I-p5+14 [45]. A sample of each was placed into a tared, plastic vial, weighed and the ^125^I radioactivity measured using an automated Wizard 3 gamma counter (1480 Wallac Gamma Counter, Perkin Elmer). The biodistribution data were expressed as % injected dose/g tissue (%ID/g). In addition, samples of each tissue were fixed in 10% buffered-formalin for 24 h and embedded in paraffin for autoradiography.

### 3.9. Micro-Autoradiography and CR Staining

For autoradiography, 6- μm-thick sections were cut from formalin-fixed, paraffin-embedded blocks, containing tissues from mice that had received ^125^I-p5+14. Mice were euthanized at 1 h pi of radiotracer. The sections were placed on Plus microscope slides (ThermoFisher Scientific, Waltham, MA, USA), dipped in NTB-2 emulsion (Eastman Kodak, Rochester, NY, USA), stored in the dark and developed after a 4 day exposure. Each section was counterstained with hematoxylin and eosin. Detection of amyloid was achieved in consecutive tissue sections by staining with an alkaline Congo red solution (0.8% *w*/*v* Congo red, 0.2% *w*/*v* KOH, 80% ethanol) for 1 h at room temperature followed by counterstain with Mayer’s hematoxylin for 2 min. Blinded qualitative assessment of amyloid load in Congo red-stained sections of liver and spleen was performed by an experienced amyloid researcher who ranked each tissue on a 0 to 4+ scale. All tissue sections were examined using a Leica DM500 light microscope (Leica, Wetzlar, Germany) fitted with cross-polarizing filters (for Congo red). Digital microscopic images were acquired using a cooled CCD camera (SPOT; Diagnostic Instruments, Sterling Heights, MI, USA).

### 3.10. Peptide Histochemistry

Peptide p5+14 (with a cysteine residue at the N-terminal) was prepared for tissue staining by either biotinylation, according to the manufacturer’s instructions, using a maleimide-biotin conjugation kit (Pierce, Grand Island, NY, USA), or for overlay-micoautoradiography by radioiodination with ^125^I, as described above. Six μm-thick formalin-fixed, paraffin-embedded human amyloid-laden tissue sections were deparafinized placed on slides and incubated in citrate antigen retrieval solution (Citrus Plus; BioGenex, Fremont, CA, USA) at 90 °C for 30 min, and then, biotinylated-peptide p5+14 was added at a concentration of 5 μg/mL in PBS and incubated overnight at 4 °C in a humidified chamber. The slides were developed using the Vectastain Elite ABC development kit (Vector Labs, Burlingame, CA, USA) and visualized using diaminobenzidene (Vector Labs). Alternatively, ^125^I-p5+14 (~5 ng/mL) was placed on the tissue sections, incubated at room temperature for 1 h, before being developed as described above (Section 3.9).

### 3.11. Human AL and ATTR Extract Preparation

Human amyloid tissue extracts were prepared using the water flotation method as described by Pras *et al.* without modification [66]. Purified amyloid material isolated in the water wash was collected and lyophilized before use.

### 3.12. Peptide ex Vivo Amyloid Binding Assay

Twenty-five microliters of 1 mg/mL AL extract, or synthetic rVλ6Wil variable domain fibrils [55] were centrifuged in a 0.5 mL microfuge tube at 21,000× *g* for 5 min. The supernatant was discarded and pellet resuspended in 200 µL of PBS with 0.05% Tween-20 (PBST). Ten microliters of a 1:100 dilution of ^125^I-p5+14 (~100,000 counts per minute (CPM); ~5 ng peptide) stock was added to the suspension. The mixture was rotated at RT for 1 h. Samples were then centrifuged twice at 15,000× *g* for 10 min. Supernatants and pellets were separated after each step and the radioactivity in each was measured using a Cobra II gamma counter (Perkin Elmer) with a 1 min acquisition. The percentage of ^125^I-p5+14 bound to pellet was determined as follows:

% Bound = Pellet CPM/(Pellet CPM + Supernatant CPM) × 100

To demonstrate the effect of increasing ionic strength on the peptide-amyloid fibril interaction, the peptide binding assay was performed, as above, in solutions of 10 mM phosphate buffer (pH 7.6) containing 0.15 (PBS), 0.25, 0.5, 1.0 or 2.0 M NaCl.

### 3.13. Binding of Biotinylated p5+14 to Synthetic rVλ6Wil Fibrils by EuLISA

Synthetic rVλ6Wil fibrils formed by agitation of a 1 mg/mL solution [67] were coated onto wells of NUNC Maxisorb plates (8 µg/mL, 50 µL) and the plates incubated overnight at 37 °C. The dried wells were washed ×1 with assay buffer (PBS containing 0.05% Tween 20) blocked with 200 µL per well of 1% BSA (*w*/*v*) in PBS at 37 °C for 1 h, followed by addition of biotinylated p5+14 diluted over the range 1 × 10^−10^–1 × 10^−6^ M. To determine background, triplicate wells lacking amyloid fibrils were used that contained only the peptide. After a 1-h incubation at 37 °C, the wells were washed ×2 and a 1/1000 dilution of europium/streptavidin (Perkin Elmer) added for 1 h before being developed with Perkin Elmer enhancement solution. Bound europium was measured by using a Victor 3 Wallac microplate reader (Perkin Elmer). The data were analyzed using SigmaPlot (SPSS: An IBM Company, Armonk, NY, USA)

### 3.14. Computer- Modeling of Peptide Docking to Amyloid Fibrils

For the docking studies, the ZDOCK server was used to generate 100 theoretical models of the interaction of p5+14 with amyloid fibrils composed of Aβ(17-42) (PDB # 2BEG; [68]) ZDOCK searches all possible binding modes in the translational and rotational space between the two proteins and evaluates each pose using an energy-based scoring function. The lowest energy p5+14 and Aβ(17-42) fibril complex was first solvated in a TIP3P water box, and then subjected to 20 ns of molecular dynamics (MD) simulation using NAMD 2.9 [69] with the CHARMM C36 force field [70]. In the simulation, the van der Waals interaction was smoothly turned off between 8.5–10 Å using a switching function. Long-range electrostatic interactions were treated using the Particle-Mesh Ewald (PME) method [71] with a 1.0 Å grid spacing. The time step for integration was 1 fs. Langevin dynamics was used to maintain a constant temperature at 310 K, while the Nosé-Hoover Langevin-piston algorithm was used to maintain a constant pressure at 1 bar.

The molecular mechanics-Poisson Boltzmann/surface area (MM-PB/SA) method [72,73] was used to compute the binding free energy of the peptide with the Aβ(17–42) fibril. The total binding energy ΔG*_bind_* was defined as ΔG*_bind_* = G*_complex_* − G*_receptor_* − G*_ligand_*. Each free energy term consisted of the gas phase MM energy (ΔE*_gas_*), the solvation free energy (ΔG*_sol_*), and the vibrational entropy contributions (*T*Δ*S*). ΔG*_sol_* was estimated from the PB theory and solvent accessible surface area (SASA) calculations which yielded ΔG*_polar_* and ΔG*_nonpolar_*. In the PB, energies were evaluated at 0.1 M, and 1.0 M NaCl concentrations to interrogate the effect of ionic strength on the electrostatic interactions between p5+14 and the Aβ fibril. A surface tension coefficient (γ) of 0.0072 kcal/(mol·Å^2^) was used to calculate the nonpolar solvation free energy contribution. Due to its prohibitive computational cost and the inherent difficulty in determining accurate absolute entropy for large protein-peptide complex systems, the vibrational entropy contribution was not included in our calculation. E*_gas_* and ΔG*_sol_* were computed for 1000 snapshots extracted evenly from the last 2 ns of the MD trajectory.

## 4. Conclusions

Amyloid deposits contain a relatively unique form of hypersulfated proteoglycans that can be specifically targeted using synthetic, heparin-binding, polybasic peptides such as p5+14. The efficient targeting of amyloid deposits affords the potential for the development of new diagnostic and therapeutic agents for these diseases.

We have developed and characterized radiolabeled p5+14 that preferentially binds to amyloid deposits in a murine model of systemic AA amyloidosis, as well as in amyloid-laden tissue specimens and extracts from patients with AL and ATTR. Remarkably, the peptide was not retained in healthy, amyloid-free tissues as evidenced by SPECT imaging and micro-autoradiography—another feature of the p5+14 peptide that supports its use as an agent for amyloid detection using molecular imaging. *In vitro* binding assays demonstrated that, in addition to the hypersulfated GAG moieties in amyloid, p5+14 also bound a common motif on amyloid fibrils with high affinity via electrostatic interactions. Molecular dynamics simulations predicted possible docking mechanisms of the p5+14 peptide with an archetypal Aβ(17–42) amyloid fibril and confirmed that charge-charge interactions were critical.

Our findings support the evaluation of radiolabeled p5+14 for amyloid imaging in the clinical setting. Translation of this reagent toward clinical trial is ongoing under the aegis of the Science Moving towArds Research, Translation, and Therapy (SMARTT) Program at the National Heart Lung and Blood Institutes (NHLBI) at the National Institutes of Health. Introduction of a novel whole body imaging protocol into patient care in the US would provide physicians with a quantitative distribution amyloid burden that could prove invaluable for diagnosis, prognostication, patient stratification, and monitoring response to therapy. Ultimately, this capability may improve outcomes and survival for patients with these devastating diseases.
